# AppER: Design and Validation of a Mobile Application for Caregivers of Patients with Duchenne Muscular Dystrophy and Their Families in Spain and Latin America

**DOI:** 10.3390/muscles4040043

**Published:** 2025-10-10

**Authors:** Jaume Barrera, Imanol Amayra, David Contreras, Alicia Aurora Rodríguez, Nicole Passi, Javiera Ortega, Óscar Martínez

**Affiliations:** 1 Neuro-E-Motion Research Team, Department of Psychology, Faculty of Health Sciences, University of Deusto, 48007 Bilbao, Spain; 2 Department of Telematics Engineering, Pontificial University of Comillas, 28015 Madrid, Spain; 3 Center for Research in Psychology and Psychopedagogy (CIPP), Faculty of Psychologyy and Psychopedagogy, Pontifical Catholic University of Argentina, Buenos Aires 1107, Argentina; javiera_ortega@uca.edu.ar

**Keywords:** mHealth, Duchenne muscular dystrophy, informal caregivers, content and face validation, psychosocial profile, quality of life, level of dependence

## Abstract

Aim: The study developed and validated AppER, an mHealth tool for informal caregivers of children with Duchenne Muscular Dystrophy, and examined differences between app users and non-users. Methods: Four phases were followed: (1) focus groups with experts and caregivers to identify care-related domains; (2) prototype development and validity testing (CVR, I-CVI, I-FVI) using the MARS scale; (3) implementation of the final app; and (4) psychosocial profiling of 88 caregivers (42 users and 46 non-users) measuring quality of life, dependency, somatic symptoms, and coping strategies. Results: AppER showed high content and face validity, surpassing reference thresholds. In the psychosocial analysis, users reported lower perceived quality of life than non-users, despite no significant differences in dependency, somatic symptoms, or coping strategies. Conclusions: Employment patterns differed: more users were dedicated to household tasks, while more non-users were self-employed, suggesting economic factors may affect app uptake and quality of life perceptions. Findings indicate AppER is a valid, well-rated support tool, and that caregivers with lower perceived quality of life may be more inclined to adopt digital health solutions, potentially to address complex care demands. Designing targeted digital interventions may be particularly valuable for those in less favorable socioeconomic contexts. Because of the small sample and between-group imbalances, results are exploratory and warrant confirmation in larger, balanced samples.

## 1. Introduction

Duchenne muscular dystrophy (DMD) is a rare X-linked genetic disease [1], with a global prevalence of 7.1 cases per 100,000 males and 2.8 per 100,000 in the general population [2]. This condition, caused by a mutation in the dystrophin gene, primarily affects males and leads to progressive muscle degeneration and increasing functional dependence that worsens with age [3,4].

Due to its low prevalence, high geographic dispersion, and remoteness from clinical centers, access to resources and specialized services is often limited, particularly in large regions as in Latin America [5,6,7,8].

Muscle weakness usually appears in early childhood, with loss of ambulation ability between the ages of 8 and 14 [9,10,11,12,13]. In adolescence, it leads to severe motor disability and complications such as scoliosis, osteoporosis, respiratory and cardiac involvement, often requiring assisted ventilation. Ultimately, it leads to cardiorespiratory death [4,14,15,16,17,18,19,20].

Since DMD has no cure, most patients require constant assistance from their family caregivers, who provide emotional support, assistance with basic feeding, dressing, hygiene and mobility activities, as well as coordination of medical treatment [16,21,22]. This imposes a considerable emotional, physical, and financial burden [23].

Previous studies have shown that caregivers of children with DMD experience high levels of stress and emotional burden, affecting their well-being and quality of life [21,24,25,26,27,28,29,30]. The responsibility of meeting the patient’s physical, emotional, and medical needs, along with managing multiple treatments, significantly impacts family dynamics, interpersonal relationships, financial aspects and the caregiver’s professional development [16,21,23,31]. Caregivers also adjust their coping strategies over time to manage stress and emotions, with effectiveness varying across individuals [32,33,34,35].

These differences in coping strategies are particularly significant when considering the specific emotional impact faced by caregivers, especially mothers. Aware of the potential genetic transmission, they may feel responsible and engage in self-blame as the disease progresses. These high levels of stress ultimately culminate in a greater vulnerability to somatic symptoms, reporting more severe symptoms than male informal caregivers, in addition to also showing greater anxiety, depression and social isolation [21,23,26,36,37,38]. Both parents may experience disbelief, denial, distress, guilt, fear, confusion, and helplessness in the initial stages [38,39,40].

In this context of high emotional and physical demands, exacerbated by geographic dispersion and isolation that many families face, electronic health tools (eHealth) have emerged as a promising alternative to reduce the load on caregivers. Examples include online telecare programs aimed at both patients with neuromuscular diseases (NMDs) [41,42,43] and family members [44]. Another example is mobile health (mHealth): these digital applications, accessible via smartphones and other devices, can facilitate patient monitoring, organize treatment management, and improve communication with healthcare professionals [45,46]. Additionally, they can offer educational resources, personalized reminders, and access to support networks, thereby reducing the feeling of isolation and improving caregivers’ quality of life [47,48,49].

Recent reviews have evaluated how mHealth can influence the health and well-being of caregivers of people with rare diseases (RD) [50,51]. Although the high frequency of phone use for social media and messaging could facilitate the adoption of these tools, their effectiveness remains conditioned by factors such as usability, interface design, and lack of customization to the specific needs of caregivers of children with DMD [52].

Although mHealth tools for chronic diseases are available, the overwhelming majority of applications are patient-oriented and address issues related to medical information, treatment reminders, and the implementation of emergency measures. Little attention is paid to family caregivers’ informational and psychosocial support needs. While some applications explicitly focus on caregivers’ emotional well-being and provide psychosocial support, coverage remains insufficient, showing that fundamental needs such as stress management and caregiver burden remain unmet [26,33]. AppER addresses this gap by offering a caregiver-centered mobile health solution for DMD that integrates psychosocial support features alongside care management, going beyond the functionality of current applications.

Despite the potential of mHealth, there is still little evidence regarding its real clinical impact on caregivers’ well-being, and adherence to such tools remains a challenge [48,53]. Furthermore, no studies have analyzed in detail the characteristics of caregivers who use these applications compared to those who do not. Previous research has observed that informal caregivers of children with neuromuscular diseases tend to care for patients with a moderate to severe level of dependence, suggesting that they could be the most likely users to benefit from these technologies [21,43].

To our knowledge, no study has specifically validated a mobile application to support caregivers of children with DMD incorporating both patient and caregiver perspectives. Patient-reported outcomes (PROs), including patient-centered PROs (PC-PROs), capture symptoms, quality of life, and treatment impact directly from patients and can enhance clinical communication; their development typically follows iterative content and face validity phases [54,55,56,57,58,59,60]. In technological applications, face validity also refers to the sensory characteristics (color, sound, etc.) with which the information is presented. This validation process involves both content experts and lay experts, combining their opinions to form the PRO measurement: content experts evaluate theoretical accuracy and relevance, while lay experts provide input on comprehensibility and usability [61,62,63,64].

The aim of this study is to develop and validate an mHealth application for caregivers of children with DMD. A mobile application specifically designed for this population could offer tools for treatment and medication management and reminders, tracking disease progression, access to DMD educational information, monitoring symptoms and health records, and connecting with support networks and other caregivers [50,51,52,65,66]. In addition, following the PC-PRO model criteria, content and lay expert assessments will be included to determine the content and face validity of the instrument. Finally, the psychosocial profile of informal caregivers of DMD patients who choose to use the designed application compared to those who do not use it will be established.

## 2. Results

### 2.1. Step 1. Design of the Initial Focus Group

During the group sessions, participants mentioned domains regarding caregivers’ needs and daily challenges and discussed different aspects of transforming them into app content. Among them are service access, daily tasks organization, caregiver well-being tracking, and DMD training.

In the AppER development phase, the 12 participants 4 male (33.3%) and 8 female (66.7%) had a mean age of 39.22 (SD = 7.74). During the three focus group sessions, the five domains for improving the care provided to children with DMD were discussed: Health Services, Technological Aids, Time of Care, Caregiver Health Care, and Education. These were identified according to the needs mentioned by the lay experts and content experts. Discussions on each domain were conducted on the following aspects: access optimization, time reorganization, caregiver health care, and DMD training. The resulting discussion and debate identified the benefits that should be prioritized in AppER:

Healthcare: tools to track symptoms, schedule medical appointments and improve communication with healthcare professionals.

1. Technical aids: resources for managing assistive devices and mobility aids required by the patient.
2. Time dedicated to caregiving: functions to organize daily tasks, medication schedules and caregiving responsibilities.
3. Caregiver’s own health care: options for monitoring the caregiver’s well-being, managing stress and accessing support networks.
4. Training received: educational resources to enhance caregivers’ knowledge of DMD, medical procedures and best practices.

### 2.2. Step 2. Design of the Prototype Application and Determination of Content and Face Validity

After the identification of the key domains, the validation of the content and appearance of the application was carried out with the participation of 27 lay experts; 24 (88.9%) were female and 3 (11.1%) were male. These had a mean age of 41.59 (SD = 8.51). Most of the sample was from Mexico (N = 13; 48.1%), Chile (N = 6; 22.2%), Ecuador (N = 5; 18.5%), and other Latin American countries (N = 3; 11.1%; Argentina y Guatemala). Content validity was assessed by applying the CVR and the I-CVI. For face validity, the I-FVI was applied.

The use of the MARS scale allowed the experts to provide quantitative results, which facilitated the optimization of items based on user experience. The results of this evaluation made it possible to determine the relevance or not of each item of the application, which ensures that the application meets the highest standards of usability and accessibility for a specific purpose.

The results (Table 1) showed that most of the items exceeded the cutoff of 0.407 in the CVR, 0.79 in the I-CVI and 0.79 in the I-FVI, indicating adequate relevance of the items included in the application.

Based on these results, modifications were made to the design’s accessibility features included changing some dialectal words to Latin American Spanish, adding an alarm system, sending notifications, and improving the visual appeal of the headings by changing their font size and iconography; presenting medical information, adding a chat feature to facilitate communication between caregivers and professionals; adding resources like adaptability web pages and organizing shortcuts within the interface by arranging the screens in accordance with the domains. These improvements were implemented in the final version of the application prior to its implementation in the next phase of the study. In addition, video tutorials were recorded on the YouTube channel [67], teaching how to use the application: how to access the interface, change preferences and enter the main functions, among other elements, which generated additional help to achieve familiarity with AppER.

### 2.3. Step 3. Implementation of the Final Version of AppER

Once the validation stage was completed, the application was implemented in a study with 88 informal caregivers of children with DMD. The majority of sample were female (N = 84; 95.5%) and the 4.5% were male (N = 4). Participants were divided into two groups: an experimental group (N = 42), consisting of caregivers who chose to use AppER, and a control group (N = 46), consisting of caregivers who chose not to use the application.

The sample was matched in terms of age, number of children, number of people in the household, and patient age. However, in terms of gender distribution, within the experimental group, 9.5% of the participants were male (n = 4) and 90.5% were female (n = 38), while in the control group, the sample was composed exclusively of women (n = 46). Significant differences were observed in terms of the gender of the study participants (χ^2^ (1) = 4.590, *p* = 0.032) and employment status (χ^2^ (7) = 44.195, *p* = 0.000), with a higher housework in AppER users (57.1%) and more ‘self-employed’ in controls (37.1%). (Table 2).

### 2.4. Step 4. Study of the Psychosocial Profile of AppER Users

The analysis between the control group and the experimental group revealed significant differences in the CarerQoL score. The control group had a significantly higher score compared to the experimental group (Z = −3.706, U = 430.000, *p* = 0.000).

Statistically significant differences were found for the item problems in the relationship with the care recipient, with the experimental group reporting more problems (78.6%) than the control group (2.6%) (χ^2^(2) = 53.542, *p* = 0.000; V = 0.813, *p* = 0.000). The experimental group also reported greater difficulty balancing care tasks with daily activities (47.6%) compared with the control group (15.4%) (χ^2^(2) = 15.476, *p* = 0.000; V = 0.437, *p* = 0.000). The experimental group likewise reported greater difficulty receiving help from others (7.1%) than the control group (23.1%) (support domain) (χ^2^(2) = 53.542, *p* = 0.000; V = 0.813, *p* = 0.000). No differences were observed for the remaining CarerQoL items.

**Figure 1:**
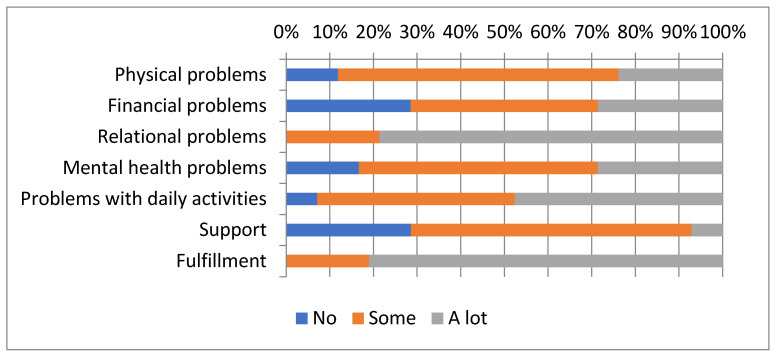
CarerQol items scores for the experimental group.

For domains without differences relative to the control group, the experimental group presented some or a lot of physical problems (88.1%), mental health problems (83.4%) and financial problems (71.5%) (Figure 1).

A univariate analysis of covariance (ANCOVA) was conducted to examine the relationship between quality of life and somatic symptomatology across the groups. No relationship was found between both variables F(1, 62) = 2.81 (*p* = 0.099).

The analysis of the Barthel Index showed that the experimental group obtained a mean score of 80.15 (SD ± 24.38), while the control group had a mean of 51.57 (SD ± 31.31). The Mann–Whitney U test indicated a no statistically significant difference in the level of dependency between the groups (Z = −0.995, U = 606.500, *p* = 0.320)

No statistically significant differences were observed in somatic symptomatology between the groups, although the trend was for the experimental group to score higher than the control (Experimental group 10.13 ± 5.16; Control group 8.41 ± 5.30). And no significant differences were found between the types of coping either (Table 3).

## 3. Methods

The present study was divided into two phases. The first aimed to establish content and face validity in three steps. The second phase consisted of the fourth step, aimed at establishing the profile of informal caregiver users. The first step consisted of the identification of the domains to be considered for the application and relied on focus groups with caregivers and DMD experts. Subsequently, in the second step, an initial prototype of AppER was built and a content validity analysis was performed. The third phase was the implementation of the final version of AppER with the improvements derived from the previous phase. The fourth and last phase consisted of the psychosocial profile comparison between non-professional caregivers of patients with DMD who were AppER users and those who were not AppER users.

### 3.1. Step 1. Design of the Initial Focus Group

In the first phase of the study, information was gathered on the needs of caregivers in relation to the challenges they face on a daily basis. Therefore, focus group were organized with the participation of caregivers and health care professionals with experience in DMD.

The focus group was composed by seven content experts, including three psychologists with experience in NMD, two clinical professionals specialized in NMD and two computer scientists; and five lay experts, members of the Asociación de Enfermos Neuromusculares de Bizkaia (BENE) Federación Española de Enfermedades Neuromusculares (ASEM). To avoid any bias, we chose to gather as many members as possible in this focus group.

Based on the focus group recruitment guidelines of Hollis et al. [68] and Ivanoff and Hultberg [69], participants were selected using convenience sampling, ensuring diversity in caregiving experience and professional expertise in neuromuscular diseases. Five focus group sessions, each lasting one hour, were conducted from March to May 2019 in a structured session design. The data collected in sessions were transcribed and analyzed with a focus on thematic content using the methodology of Braun and Clarke [70]. In this case, common patterns in the participants’ discourses were identified, and these mentions were grouped under the five largest categories. As a result, for this phase, the transcription of the discussions was obtained, the coding of the themes occurred, and the information was organized according to the five identified domains.

### 3.2. Step 2. Design of the Prototype Application and Determination of Content and Face Validity

Based on the results of the previous phase, an initial prototype of AppER was developed, incorporating the domains and functionalities identified in the focus group. In this second step, we propose to validate the contents resulting from the Focus Group and establish the degree of acceptance of their visual presentation (shape, color, icons, sound) through face validity.

The number of evaluators who participated in the content validity study was determined according to the guidelines provided by Lynn [71] and Romero Jeldres et al. [72]. A total of 27 lay experts (N = 27) were selected. The lay experts were caregivers of children with Duchenne muscular dystrophy who were members of the associations Duchenne Chile Foundation, Enlace Duchenne/Becker Muscular Dystrophy Association AC, Latin American Duchenne/Becker Muscular Dystrophy Coalition, Research Center in Psychology and Psychopedagogy, Pontifical Catholic University of Argentina, Buenos Aires, Argentina, chosen for their direct experience in managing the disease and their ability to evaluate the usability and relevance of the app content. The participants were from Mexico, Chile, Ecuador, Argentina and Guatemala.

The inclusion criteria included being an adult over 18 years of age; being an informal caregiver, relative of a child with a diagnosis of DMD; already having previous experience in the care of a patient with DMD; using Spanish as one of the main languages of communication between caregiver and professional; and providing written informed consent. The exclusion criteria included the presence of other previous medical or psychiatric diagnoses unrelated to DMD; uncorrected sensory deficits that may interfere with the assessment; and functional illiteracy that precludes proper use of the application.

The 27 lay experts rated AppER using the Mobile App Rating Scale (MARS) [73]. They assessed the content validity of AppER according to the dimensions of engagement, and the face validity based on the dimensions of aesthetics and functionality. To evaluate the content validity, the criteria of Lawshe [74], Lynn [71] and Zamanzadeh et al. [64] will be applied, using a five-point Likert-type scale to assess the relevance of each item. Based on the responses, the Content Validity Ratio will be calculated from the following equation:

$$CVR=\frac{N_{E}-N/2}{N/2}$$

The numerical value of the CVR according to Lawshe’s table for the 27 panelists was 0.407 [72,74].

The Content Validity Index per Item will be calculated using the following formula:

$$I-CVI=\frac{Raters\;in\;agreement}{Number\;of\;total\;raters}$$

And the Face Validity Index will be calculated using the formula:

$$I-FVI=\frac{Raters\;in\;agreement}{Number\;of\;total\;raters}$$

Following Yusoff’s recommendations [75,76], a value greater than or equal to 0.79 was considered valid.

In this phase of the study, participants analyzed the functionalities of the application and the relevance of the content presented. During the evaluation period, users employed AppER to record their needs and activities, as well as to obtain information about the medical resources and associations involved. They were subjected to a structured test session, through which they systematically explored each section of the application and completed a more detailed assessment of whether the application was easy to use, whether the information presented was clearly readable, and whether or not the functionalities were useful. They all evaluated their understanding of the instructions, identified the main functionalities of the application, the control options available and the usefulness of the educational content.

### 3.3. Step 3. Implementation of the Final Version of AppER

After evaluating content validity in the previous phase, the final implementation of AppER was carried out, incorporating improvements derived from the analysis of the obtained results. This phase aimed to consolidate the application as an effective tool aligned with the real needs of caregivers of children with DMD.

The AppER application (Appendix A) was designed as a digital tool in an agenda format, intended to guide and remind caregivers, both informal and formal, of the patients’ daily needs and activities. The different sections included are shown in Table 4.

Different tutorial videos were also published in two YouTube channels led by project collaborators in Chile and Spain [67], which offer detailed instructions on the installation and use of AppER on Android devices.

### 3.4. Step 4. Study of the Psychosocial Profile of AppER Users

The study involved 88 informal caregivers of children with DMD (n = 88), recruited through various patient associations in Spain and Latin America, including Fundación Duchenne Chile, Enlace Distrofia Muscular Duchenne/Becker, Asociación de Distrofia Muscular Argentina, Hospital Clínico San Borja Arriarán, Coalición Latinoamericana Duchenne/Becker and the Federación Española de Enfermedades Neuromusculares.

The participants were divided into two groups: an experimental group (n = 42), composed of caregivers who decided to use AppER, and a control group (n = 46), composed of those who chose not to use the application. The purpose of this analysis was to determine the psychosocial profile of the caregivers. The data were kept matched by age to ensure comparability between groups in this aspect. However, data were not kept for gender or level of patient dependency.

The inclusion criteria applied were being over 18 years of age; being an informal caregiver (family member or close friend) of a child diagnosed with DMD; providing written consent to participate in the study; and using Spanish as one of the main communication languages. The exclusion criteria were the presence of any other medical diagnosis unrelated to NMD; presence of psychological or psychiatric diagnoses not secondary to NMD; uncorrected sensory deficits that would affect completing the evaluation protocol; and illiteracy.

### 3.5. Instruments

#### 3.5.1. Sociodemographic Questionnaire

A questionnaire was designed through the Qualtrics platform to collect sociodemographic and socio-educational data. First, caregiver data were collected: age, sex, marital status, number of children, people living at home, nationality, and current residence. Subsequently, data about the person being cared for were requested: type of illness and age. Finally, socio-educational data of the caregiver were collected, including years of education and employment status.

#### 3.5.2. CarerQol

This is an instrument created by Brouwer [77] and adapted into Spanish by Ruiz Reverte [78], evaluating care-related quality of life. It is divided into two parts: On one hand, the CarerQol-VAS, which measures caregivers’ well-being in terms of happiness using a visual analog scale (VAS) from 1 to 10, where 1 represents “completely unhappy” and 10 is “completely happy” [78]. On the other hand, the CarerQol-7D, which evaluates the subjective burden of caregiving through 7 questions covering dimensions such as mental and physical health, relationship problems, and satisfaction with caregiving [77]. The tool has a Cronbach’s alpha of 0.62 [79], which is considered a good internal consistency for research [80,81].

#### 3.5.3. Barthel Index

Developed by Mahoney and Barthel [82] and adapted to Spanish by Baztán [83], it assesses the patient’s independence in 10 basic activities of daily living. Each activity is scored from 0 to 15, with a total score ranging from 0 to 100 or from 0 to 90 if the patient uses a wheelchair. Scores classify the patient’s dependence, from total dependence (<20 points) to total independence (100 points). The index has shown high reliability (*p* < 0.80) and concurrent validity, with significant correlations found between Barthel and other measures, such as the Katz Index for daily living activities (*p* = 0.70–0.80) and the Functional Independence Measure scale (*p* = 0.80–0.90) [84,85,86].

#### 3.5.4. COPE-28

The Spanish version of Carver’s Brief COPE [87], based on the theory of Lazarus and Folkman [88] and translated into Spanish by Morán et al. [35], was used. It is a 28-item inventory divided into 14 subscales, evaluating effective coping styles (such as active coping, planning, and social support) and ineffective (such as self-distraction, venting, and denial). A Likert scale of 0 to 3 points is used. The inventory’s internal consistency is 0.78 [89].

#### 3.5.5. PHQ-15

The Patient Health Questionnaire-15 (PHQ-15), created by Kroenke [90] and validated in Spanish by Montalbán [91], assesses the severity of somatic symptoms and somatization. It consists of 15 items representing 90% of common somatic complaints in primary care. Patients rate each symptom on a scale from 0 to 2, and the total score determines the severity of symptoms. The tool has been translated and validated in several languages, with a Cronbach’s alpha of 0.78, indicating good test–retest reliability and strong internal consistency, as well as supporting its criterion and construct validity [90,91,92].

#### 3.5.6. MARS

Developed by Stoyanov et al. [73], MARS assesses the quality of mobile health applications through 23 items, scored from 1 to 5, grouped into four objective subscales (engagement, functionality, aesthetics, and information quality) and one subjective subscale [73,93]. The total score ranges from 1 to 5, with higher scores indicating better app quality. The scale has shown high inter-rater reliability (ICC = 0.79–0.93) and strong internal consistency, with a Cronbach’s α = 0.90 for the total scale, 0.80–0.89 for subscales [93,94].

### 3.6. Statistical Analysis

To evaluate the content validity of the AppER (step 2) application, the Content Validity Ratio (CVR) and the Item Content Validity Index (I-CVI) were calculated [74,75]. For face validity, the formula for the Index of Face Validity per Item (I-FVI) [76] was applied.

In Step 4, a descriptive analysis was performed to provide a detailed summary of the main characteristics of the studied variables. Normality tests were conducted using the Kolmogorov–Smirnov test; therefore, non-parametric tests were chosen.

To compare two independent groups, the Mann–Whitney U test was applied. An analysis of covariance (ANCOVA) was also performed to compare group means. Phi and Cramer’s V were calculated in the chi-square test to assess the relationship between variables. Finally, to analyze the relationship between continuous variables, the Spearman coefficient was employed. The data analysis for this study was conducted using IBM SPSS version 21.

## 4. Discussion

The aim of this study was, on the one hand, the creation and validation of an mHealth application designed for caregivers of children with DMD, and, on the other, to determine the psychosocial profile of informal caregivers of patients with DMD who use the designed application compared to those who do not. The study was conducted in four steps: (1) initial focus group design, (2) development of the AppER prototype, (3) implementation of the final version, and (4) a pilot study on caregiver profiles and AppER usage. The focus group included seven content experts and five lay experts to define key domains. Later, 27 caregivers of children with DMD participated in the design and refinement of the application. In the final step, the study involved 88 caregivers, divided into a control group (n = 46) and an experimental group (n = 42).

### 4.1. Step 1. Design of the Initial Focus Group

The results of the focus group were necessary to identify the most important needs of caregivers of patients with DMD and to define the most suitable domains for the design of AppER. The practice of using content and lay experts allowed for both the technical and experimental perspective, contributing to a more comprehensive vision in the application’s decision-making.

The discussions showed the need to focus on five areas: healthcare assistance, technical aids, time management, caregiver well-being, and training. Among them, the need to improve communication with healthcare professionals, optimize the organization of daily tasks, and strengthen support for the caregiver’s mental and physical health stood out. The lack of accessible resources on the management of assistive devices and the need for continuous training on DMD were also identified. These results were aligned with previous studies, where focus groups have identified concerns such as the need for multidisciplinary care as well as better social and psychological support for caregivers [95].

Research conducted on quality of life in children with DMD shows generally positive perceptions of family and leisure, these being the most representative domains. However, the caregiving experience reveals intense psychological stress, which highlights the importance of improving support systems [96,97].

For this purpose, focus groups have proven to be a useful tool for identifying needs in the management of chronic diseases, such as DMD [95,98]. The methodology with participatory co-designs, involving both patients and caregivers, healthcare professionals, and digital health experts, has been effective in the development of mobile health applications [99]. These applications can include functionalities such as medication reminders, self-monitoring tools, and caregiver shift management [98].

In this first phase, participants’ opinions were gathered, establishing a solid basis for the development of the initial AppER prototype. Although the study involved a small number of experts, the results provided the necessary information to optimize the design of the application. The application design was evaluated in step 2.

### 4.2. Step 2. Design of the Prototype Application and Determination of Content and Face Validity

The results obtained from the content and face validation of AppER confirm that the application meets criteria for usability, accessibility, and relevance for caregivers of children with DMD. The evaluation, conducted by 27 lay experts, allowed for the analysis of different aspects of the application using established metrics to assess its adequacy and functionality.

The data obtained confirmed that most items exceeded the required cutoff in the CVR, I-CVI and I-FVI. Regarding content validity, the app was positively evaluated for its credibility, the quality of the information provided, and its potential to enhance users’ knowledge about the disease and its care. This confirms that the elements included in the application are perceived as relevant and aligned with the needs of caregivers. In face validity, the quality of the presentation and graphics was rated positively. The application presented the content in an attractive way, with a high level of interactivity, and was fast and accurate. This indicates that AppER has been well designed in terms of appearance and reliability.

The validation of mobile health applications is a necessary process to guarantee their usability and quality of content and appearance [100]. Instruments such as the CVR, I-CVI and I-FVI have been used to evaluate different aspects of these digital tools, confirming that the quality of the design and the accuracy of the information are key elements in their development [101]. However, content validation alone does not guarantee the effectiveness of the application in practice, so it is necessary to evaluate the impact of AppER on care management and caregivers’ quality of life over time.

Recent research highlights the importance of including both healthcare professionals and caregivers in the validation process of applications designed for DMD [102]. While tools such as MARS have proven to be reliable for assessing the quality of e-health applications [94], it has been noted that many usability scales are geared toward technology or healthcare experts, overlooking the perspectives of end users, such as patients and caregivers [103]. This underscores the need to develop more inclusive evaluation methods that reflect the real experiences and needs of those who will use these applications in their daily lives.

Based on the results obtained and users’ perceptions, adjustments were made in accessibility, information presentation, and interface organization to optimize the final version of the application before its implementation in the next study phase. The tool was positively evaluated in terms of interaction with the tool, highlighting the ability to allow data entry and send customizable notifications; in performance, evidencing a fast and accurate execution of its functions; and in the quality of information, showing that the content is accurate and aligned with the application’s objectives.

With respect to face validity, the evaluators rated positively the visual aesthetics and the quality of the graphic elements, such as buttons, icons and menus, which they considered adequate for a satisfactory user experience [104,105,106,107,108]. However, lower scores were reported for ease of use and navigation, indicating that some users found it difficult to learn how to use the app. To solve this, video tutorials were developed and made available on YouTube [67], facilitating the understanding of its functions and improving the user experience. Beyond tutorials, we will iteratively refine navigation and task flows by reducing steps, clarifying labels, and improving accessibility to increase usability and enhance adoption.

### 4.3. Step 3. Implementation of the Final Version of AppER

Apper was developed as a digital tool that supports caregivers of children with DMD in the organization of care, access to relevant information and connecting with other caregivers. The final version, developed following the content validation process, had functionalities such as activity planning, personalized reminders, support forums and access to resources, tailored to the real users’ needs [109,110].

Previous studies with mHealth applications for caregivers have highlighted their potential in improving quality of life and reducing emotional overload, as they facilitate task organization and provide social support [26,51,111]. AppER aligns with these recommendations, as it includes functionalities that allow personalized management of daily needs and activities, as well as tools to access specific information and support resources.

The inclusion of the forum in AppER also responds to previous research findings, which suggest that mHealth applications are more effective when they include spaces for interaction between caregivers, because they can exchange experiences and coping strategies [66]. Digital tools with a focus on social support, self-care strategies and stress reduction have a positive impact on the emotional well-being of caregivers [112,113].

Although further investigation is needed on long-term use and its impact on users’ quality of life, the literature indicates that caregivers are often receptive to these types of digital solutions [114]. Literature suggests that when a tool is presented as intuitive, accessible and well-integrated into daily routines, adoption is enhanced [26]. These characteristics have been prioritized in the design of AppER.

### 4.4. Step 4. Study of the Psychosocial Profile of AppER Users

The present step aimed to determine the profile of informal caregivers of patients with DMD who used AppER compared to those who did not in the level of dependency of their children, quality of life, presence of somatic symptomatology and coping strategies.

The results showed that most caregivers were female, a trend also found in the previous literature on the care of people with DMD [21,22,43,115,116,117,118,119,120,121,122,123]. These caregivers often face greater challenges related to physical and emotional burden, which may be reflected in levels of somatic symptoms and life satisfaction [124,125,126,127].

No significant differences were found between the two groups in the level of dependence. Although some studies suggest that higher support needs drive the search for digital tools, our data did not show that pattern. These studies highlight the importance of providing personalized technology interventions to caregivers of individuals with high levels of dependency, especially in areas such as feeding, mobility, and movement [113,128].

One of the most notable findings was that caregivers who opted not to use the app reported a better perceived quality of life compared to those who used it. This result could suggest that caregivers who opt to use the app face greater challenges such as problems with the caregiver’s relationship, difficulties managing activities of daily living, or problems with their own health, which motivates them to seek additional resources [129,130,131,132,133]. The difficulties faced by caregivers are linked to a greater search for eHealth resources, especially among those who have positive perceptions of their usefulness and a higher level of digital literacy [122].

Regarding somatic symptoms, although no statistically significant differences were found between the control and experimental groups, a slight trend emerged: caregivers who chose to use the application reported greater pain in arms legs and joints, fatigue and more sleep problems [134,135,136,137]. Previous studies [138] have indicated that caregivers are likely to present elevated levels of anxiety and somatic symptoms compared to the general population. These include increased stress levels, poor physical and mental health, and deterioration in quality of life [139,140,141,142,143,144,145,146].

No significant differences in coping strategies were observed between the control and experimental groups. Previous studies on coping strategies in caregivers of children with DMD present mixed results. While there are studies that claim that caregivers facing a greater burden resort to more active or support-seeking strategies [147] or that maladaptive strategies negatively impact quality of life [148,149,150], others have found no significant differences in coping as a function of caregiving circumstances [35,151]. The similarity in coping between the groups may indicate that caregivers cope in a similar way, regardless of the use of digital tools. This finding suggests that coping style alone is not a determinant for the use of mHealth resources; other factors, such as quality of life and access to support networks, may play a more influential role.

The employment situation differed between the groups, with more domestic tasks in the experimental group and more self-employed caregivers in the control group. This suggests that different economic and time constraints determine both perceived quality of life and the use of mHealth [119,152,153,154]. Differences in employment status suggest that the adoption and sustained use of mHealth also depend on role and time constraints [155,156]. Unpaid domestic tasks involve continuous care, with a greater need for digital support but less time for interaction. Paid work reduces availability, favoring asynchronous, self-paced access and brief micro-interventions [157]. Because of this, a caregiver-centered design with simple/low-effort flows and configurable reminders can improve adoption and adherence [156,158], while supplemental financial support could further improve well-being [152,153].

Despite previous literature indicated a significant relationship between patient dependency and somatic symptoms in caregivers, where caregivers of individuals with rare diseases with more severe dependency experience a considerable impact on their physical and emotional well-being [22,115,125,146], this study’s data suggested no correlation between these variables. This discrepancy may be due to sample characteristics. Nevertheless, the consolidated evidence in the literature underscores the influence of patient dependency level on caregivers’ physical and emotional health.

Among the limitations of the study that must be considered, one is the sample size, which is relatively small (n = 88) and could limit the generalizability of the findings to other caregivers of patients with DMD. The differences between groups in terms of gender and employment status reduce comparability between users and non-users and constrain external validity. Some differences may reflect the composition of the sample, with more mothers and different employment situations, rather than effects that would generalize to other populations. To improve representativeness, strategies should explicitly engage fathers/male caregivers. Additionally, the limited statistical power may have hindered the detection of significant effects in some variables, suggesting that certain differences or relationships may not have reached statistical significance due to the number of participants rather than a true absence of effect in the general population.

Regarding quality of life, we used the CarerQoL because, at the time of data collection, no broader measure of family quality of life validated in Spanish was available. The Spanish validation of the PedsQL family impact module [26] was published after our selection of instruments. Therefore, the present results should be interpreted as an approximation of quality of life, and future research should incorporate the PedsQL FIM.

This study did not directly assess socioeconomic status or digital literacy. The entire sample, including the control group, interacted via smartphone to answer the questionnaires, which reduces variability in device access and may attenuate any association between socioeconomic status and mobile phone use. Therefore, our findings on adoption should be interpreted as conditional on smartphone ownership. Unmeasured factors such as available time, digital skills, connectivity or notification preferences could continue to modulate actual app usage.

Another aspect to consider is the use of self-reported measures, which could introduce social desirability bias. Although validated instruments were used, the inherent subjectivity of the responses could affect the accuracy of the data.

It is necessary to consider the heterogeneity of the sample in terms of cultural context. Despite the fact that all participants are Spanish-speaking, caregivers belonged to different countries, introducing variability in cultural factors, health-system organization and funding, access to health resources, and social support networks. These differences could influence perceptions of quality of life, somatic symptoms, and coping strategies, as well as mHealth adoption and engagement (connectivity stability, device availability, and operational digital literacy), potentially contributing to selection into the user group, complicating the interpretation of the results.

Future studies with larger samples and longitudinal designs would be required to confirm these findings and further explore the effectiveness of digital interventions such as the application evaluated in this study. This analysis focuses on short-term cross-sectional comparisons. Therefore, we refrain from making causal inferences and interpret differences cautiously and as a possible reflection of selection. Our organization is conducting a longer-term evaluation at 6 and 12 months, the results of which will be reported separately and will serve as the basis for future iterations of AppER. To participate, it was necessary to use a smartphone from the outset. Additionally, it would be valuable to investigate how individual characteristics of caregivers and family context influence the decision to use support tools and their psychosocial impact.

The study results suggest that caregivers who use the app may experience a higher physical and psychological burden, underscoring the importance of designing digital tools that address their specific needs. Given that these caregivers already face significant challenges, future applications should integrate features that promote emotional well-being and stress management, such as self-care and psychological support modules. Additionally, it is important to acknowledge that non-users of the app may face technological barriers or lack digital literacy.

## 5. Conclusions

This study has allowed the development and validation of AppER, an mHealth application aimed at caregivers of children with DMD, as well as the exploration of the psychosocial profile of those who choose to use the tool versus those who do not. The app, designed through a validation process by content experts and real caregivers, showed adequate levels of content and face validity, and was well rated for its usability, visual presentation, and quality of information.

It was observed that those who chose to use AppER reported a poorer quality of life and were more engaged in household chores, suggesting a lower economic capacity. However, no significant differences were found between the groups in the level of child dependency, somatic symptomatology or coping strategies. This may indicate that the choice to use the app may be more related to the perceived need for support than to differences in the caregiving situation. Given the modest sample size and initial imbalances, these findings should not be generalized beyond similar contexts until they are replicated in larger, more balanced multicenter cohorts.

## Figures and Tables

**Table 1:** CVR and I-CVI.

Item	N	Lawshe	CVR	I-CVI	I-FVI
1. Interest: Is the app interesting? Does it present its content in an engaging way to increase participation?	26				0.885
2. Personalization: Does it allow adjustments or preference selection for configuration features (e.g., sound, content, etc.)?	27				0.926
3. Interaction: Does it allow user inputs, provide feedback, and send notifications? (These functions should be customizable and not overwhelming).	27				0.926
4. Target audience: Is the app’s content (visuals, language, design) appropriate for the type of user it is intended for?	27				0.852
5. Performance: How fast/accurate are the app’s features (functions) and its components (buttons/menus)?	27				0.926
6. Ease of use: Is it easy to learn how to use the app? Are the menu labels/icons and instructions clear?	27				0.778
7. Navigation: Is moving between screens logical/accurate/appropriate/uninterrupted? Are all necessary screen links present?	27				0.741
8. Gestural design: Are interactions (taps, keystrokes, swipes) consistent and intuitive across all components or screens?	27				0.889
9. Layout: Is the layout and size of buttons/icons/menus/screen content appropriate, or does it allow zooming if needed?	27				1.0
10. Graphics: Is the quality/resolution of the graphics used as buttons/icons/menus/content high?	27				1.0
11. Visual appeal: Does the app have a good appearance?	27				0.815
12. Accuracy of app description (in the app store): Are the features mentioned in the description included in the app?	27	0.407	1.0	1.0	
13. Objectives: Does the app set specific, measurable, and achievable goals (specified in the app description or within the app itself)?	26	0.385	0.769	0.885	
14. Quality of information: Is the content correct, well-written, and consistent with the app’s objectives/themes?	27	0.407	0.926	0.963	
15. Amount of information: Is the app’s purpose explained? Is it comprehensive and concise?	27	0.407	0.778	0.889	
16. Visual information: Is the visual explanation of concepts through images/graphics/videos logical, clear, and correct?	27				0.852
17. Credibility: Does the app come from a legitimate source (specified in the app store description or within the app itself)?	26	0.385	0.923	0.962	
18. Scientific basis: Has the app been tested/verified scientifically (published in scientific literature)?	26	NA	NA	NA	NA
19. Would you recommend this app to people who could benefit from it?	27				0.963
20. In the next 12 months, how many times do you think you would use this app if it were relevant to you?	27				1.0
21. Would you pay to obtain the app?	27	NA	NA	NA	NA
22. What is your overall rating of the app?	27				1.0
23. Awareness: Is the app likely to increase caregivers’ awareness throughout the disease process?	28	0.357	0.714	0.857	
24. Knowledge: Is the app likely to increase family members’ knowledge/understanding of the disease and its care?	27	0.407	0.926	0.963	
25. Attitudes: Is the app likely to change attitudes towards improving caregivers’ quality of life?	27	0.407	0.926	0.963	
26. Intention to change: Is the app likely to increase intentions/motivation to delegate their children’s care to an external caregiver easily?	27	0.407	0.704	0.852	
27. Help-seeking: Is the app likely to encourage help-seeking to reduce caregiver burden?	27	0.407	0.778	0.889	

NA: Not Applicable.

**Table 2:** Sociodemographic data.

	Contr	Exp	Tot	χ^2^	*p*
Age	40.67 ± 8.18	42.21 ± 7.49	41.41 ± 7.83	31,235	0.404
Number of children	2.39 ± 0.90	2.51 ± 1.00	2.45 ± 0.94	1662	0.798
People living in the household	4.07 ± 1.14	4.07 ± 1.19	4.07 ± 1.16	5842	0.441
Patient’s age	12.67 ± 5.09	12.79 ± 4.26	12.73 ± 4.69	21,997	0.460
Gender				4590	0.032 *
Male	0 (0%)	4 (9.5%)	4 (4.5%)		
Female	100 (100%)	38 (90.5%)	84 (95.5%)		
Marital Status				7488	0.187
Married	22 (47.8%)	26 (61.9%)	48 (54%)		
In a relationship	10 (21.7%)	5 (11.9%)	15 (17%)		
Divorced	9 (19.6%)	2 (4.8%)	11 (12.5%)		
Separated	2 (4.3%)	3 (7.1%)	5 (5.7%)		
Single	2 (4.3%)	4 (9.5%)	6 (6.8%)		
Widowed	1 (2.2%)	2 (4.8%)	3 (3.4%)		
Nationality				78,456	0.000 *
Argentina	-	1 (2.4%)	1 (1.1%)		
Bolivia	31 (67.4%)	-	31 (32.5%)		
Chile	12 (26.1%)	2 (4.8%)	14 (15.9%)		
Colombia	1 (2.2%)	2 (4.8%)	3 (3.4%)		
Ecuador	-	3 (7.1)	3 (3.4)		
El Salvador	1 (2.2%)	-	1 (1.1%)		
Spain	-	1 (2.4%)	1 (1.1%)		
USA	1 (2.2%)	-	1 (1.1%)		
Mexico	-	32 (76.2%)	32 (36.4%)		
Paraguay	-	1 (2.4%)	1 (1.1%)		
Occupation				44,195	0.000 *
Salaried	1 (2.2%)	7 (16.7%)	8 (9.1%)		
Self-employed	18 (39.1%)	4 (9.5%)	22 (25%)		
Unpaid work	6 (13%)	-	6 (6.8%)		
Unemployed due to health issues	6 (13%)	1 (2.4%)	7 (8%)		
Unemployed for other reasons	6 (13%)	4 (9.5%)	10 (11.4%)		
Retired	6 (13%)	1 (2.4%)	7 (8%)		
Housework	3 (6.5%)	24 (57.1%)	27 (30.7%)		
Student	-	1 (2.4%)	1 (1.1%)		

* *p* < 0.05.

**Table 3:** Mann–Whitney U Test Analysis of Clinical Variables and Groups.

Clinical Variables	Group	*n*	*M*	*U*	*p*	Z
Quality of Life	Experimental Control	42 39	12.88 ± 2.39 15.28 ± 2.62	430,000	0.000 *	−3.706
Somatic Symptom	Experimental Control	36 29	10.13 ± 5.16 8.41 ± 5.30	421,000	0.181	−1.337
Level of dependence	Experimental Control	40 35	80.15 ± 24.38 51.57 ± 31.31	606,500	0.320	−0.995
Active Coping	Experimental Control	42 32	4.39 ± 1.36 4.44 ± 1.34	614,500	0.517	−0.647
Planning	Experimental Control	40 31	4.00 ± 1.56 3.90 ± 1.51	619,500	0.995	−0.006
Emotional Support	Experimental Control	42 20	3.39 ± 1.99 3.45 ± 1.92	360,500	0.364	−0.908
Social Support	Experimental Control	39 32	3.55 ± 1.87 2.53 ± 1.62	602,000	0.796	−0.258
Religion	Experimental Control	41 32	3.64 ± 2.47 4.69 ± 1.80	488,000	0.051	−1.952
Positive Reappraisal	Experimental Control	42 32	4.45 ± 1.75 4.09 ± 1.40	640,500	0.725	−0.352
Acceptance	Experimental Control	42 35	4.82 ± 1.44 4.89 ± 1.07	709,000	0.780	−0.280
Denial	Experimental Control	41 28	1.27 ± 1.79 2.00 ± 1.80	500,500	0.347	−0.940
Humor	Experimental Control	39 28	1.70 ± 1.79 1.89 ± 2.00	429,500	0.119	−1.561
Self-Distracting	Experimental Control	42 31	3.39 ± 1.87 3.19 ± 1.83	619,000	0.718	−0.361
Self-Blame	Experimental Control	40 37	2.06 ± 1.69 2.45 ± 1.76	534,000	0.309	−1.017
Disconnection	Experimental Control	40 30	0.88 ± 1.11 1.27 ± 4.48	515,500	0.285	−1.070
Venting	Experimental Control	40 39	2.12 ± 1.78 2.21 ± 1.74	579,500	0.995	−0.006
Substance use	Experimental Control	38 24	0.48 ± 0.87 0.42 ± 0.830	452,000	0.939	−0.077

* *p* < 0.05.

**Table 4:** AppER: Functionality.

Functionality	Description
AppER Test	Direct access to the Qualtrics platform to complete questionnaires that assess the app’s impact on quality of life.
Resources	Provides information on associations and other useful resources depending on the user’s country.
Basic needs	Lists daily needs scheduled by the user, specifying the time for each one
Create need	Allows the user to customize new needs, indicating name, type, scheduled days, times, and special considerations.
Activities	A list of daily activities scheduled by the user, with specific times.
Create activities	Allows the user to add new activities, specifying name, type, scheduled days, times, and additional notes.
My Profile	Contains personalized data such as name, email, patient’s birthdate, country of residence, patient’s disease, and additional disease details.
Forum	Facilitates communication between caregivers to exchange experiences, with additional content provided by experts.
Report error	Allows users to report potential app issues and provides a quick solution.

**Scheme A1:**
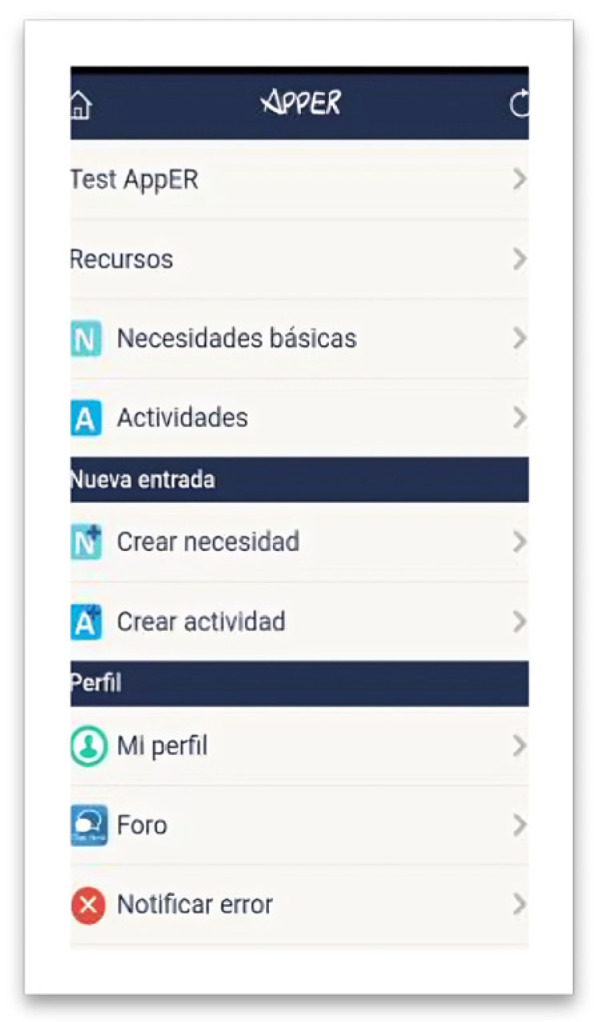
AppER Main Menu.

**Scheme A2:**
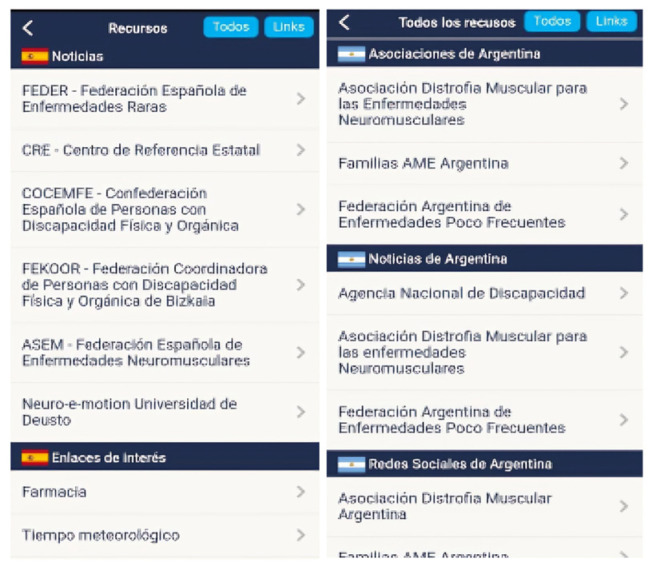
Associations and resources by country.

**Scheme A3:**
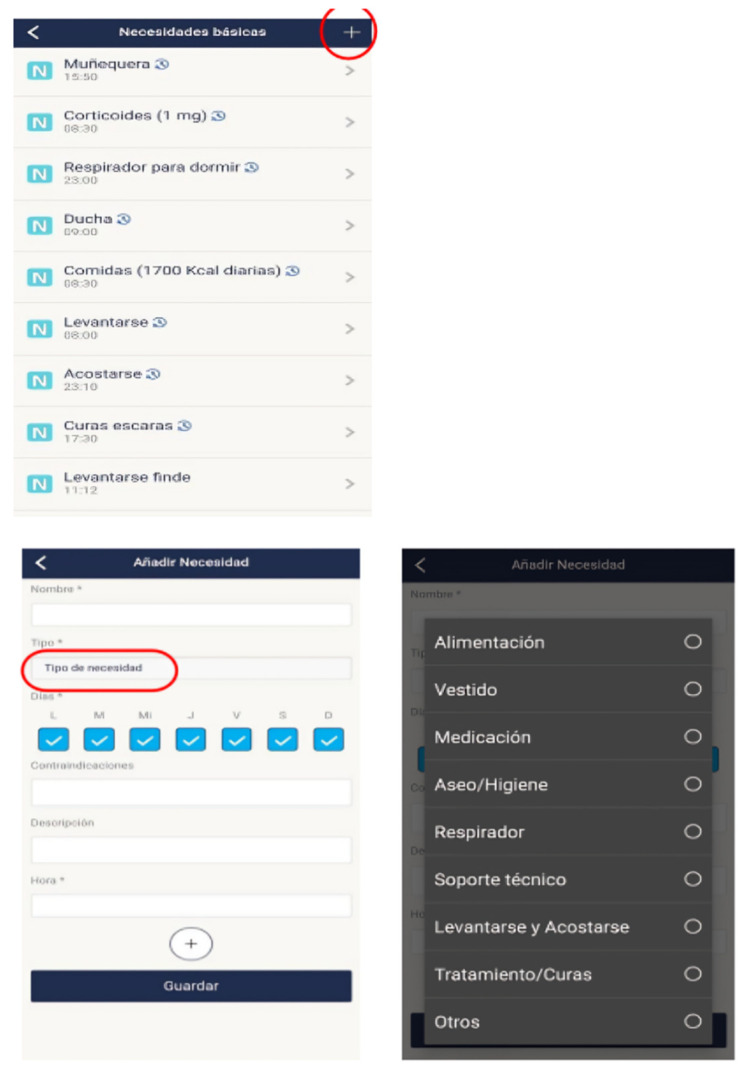
Basic needs: This section lists the day-to-day needs the user has created, along with the scheduled time for each.

**Scheme A4:**
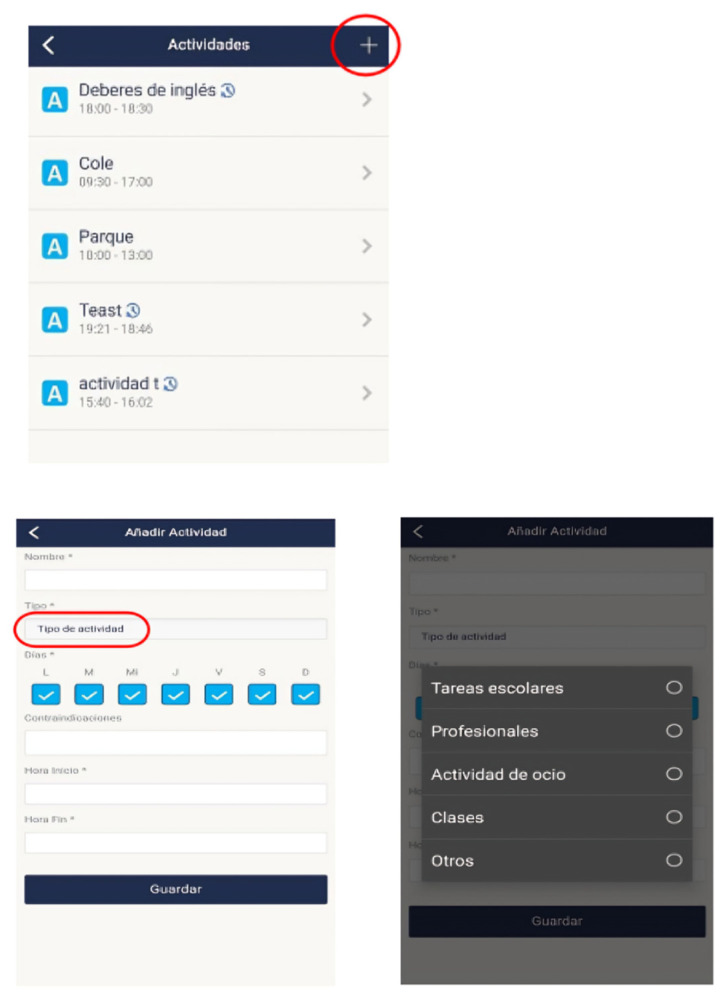
Created activities: User-created activities and times.

**Scheme A5:**
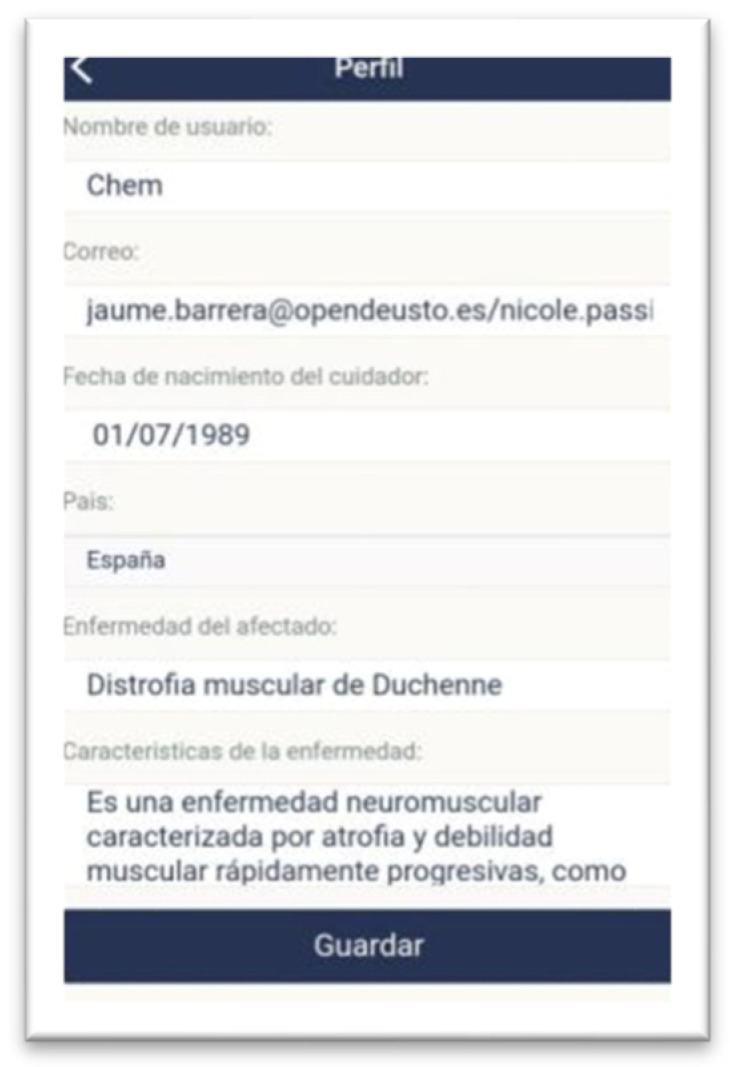
My profile: Name, email, caregiver DOB, country, diagnosis and optional condition details.

**Scheme A6:**
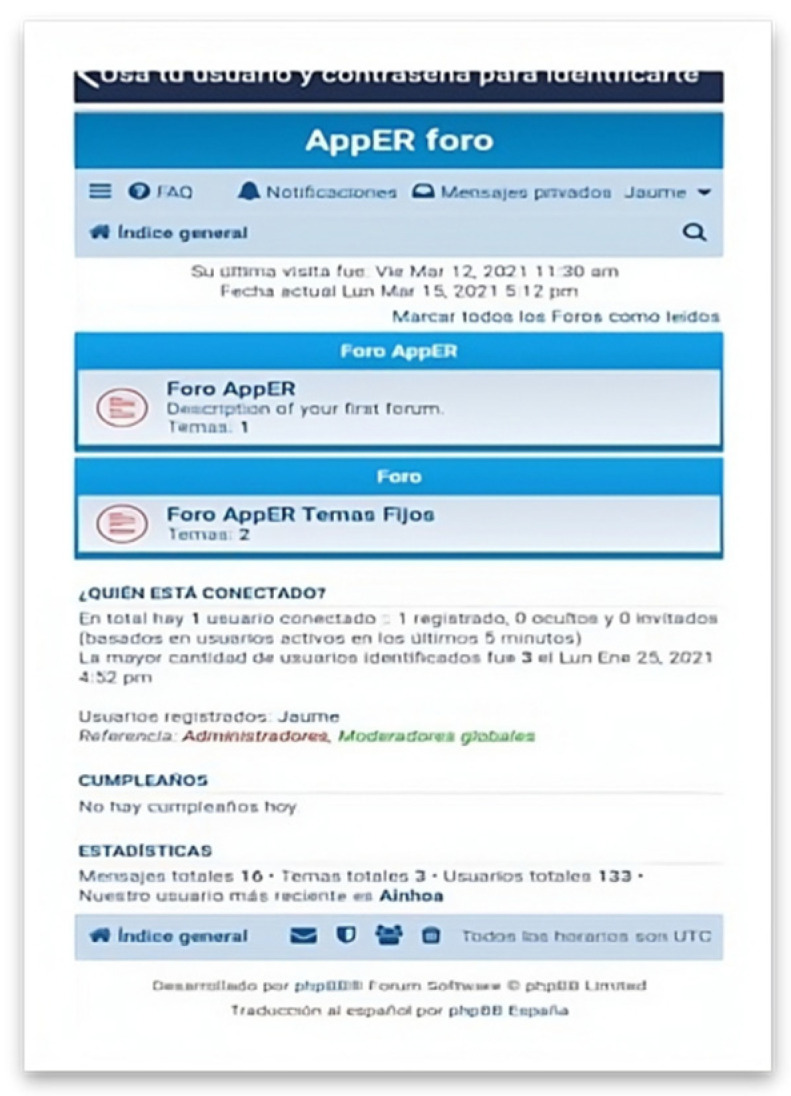
Foro AppER: Space for caregivers to connect and exchange knowledge. Pinned topics include programs and expert-reviewed guides.

**Scheme A7:**
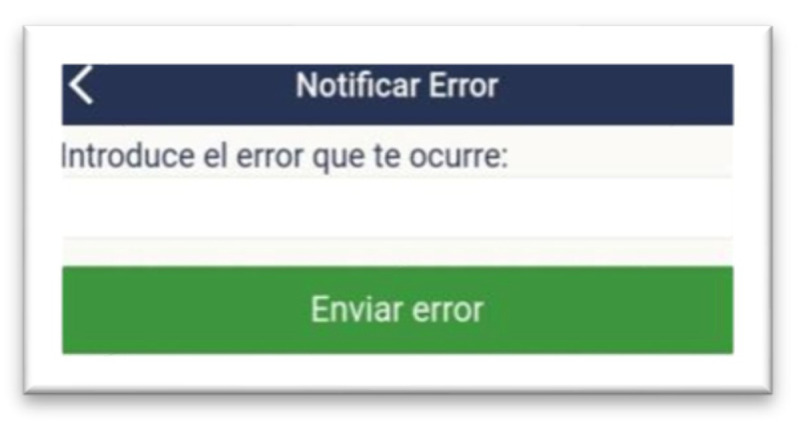
Report error: Report any app issue so it can be addressed promptly.

## Data Availability

The datasets generated and analyzed during the current study are available from the corresponding author, Jaume Barrera, upon reasonable request.
